# A Few Thoughts Concerning the Treatment of the Lower Bicuspid Teeth

**Published:** 1877-12

**Authors:** A. W. Harlan

**Affiliations:** CHICAGO, ILL.


### ARTICLE IV.

### *A Few Thoughts concerning the Treatment of Lower Bicuspid Teeth.*

BY A. W. HARLAN, CHICAGO, ILL.

Considering the fact that a large number of lower first molar teeth are lost prior to the thirtieth year; and partly in consequence of this, but more particularly, because of an imperfect development of the second molars, they too are lost; the conservation of the lower bicuspids becomes a question of importance; and as will be seen further along, of so much importance, that I am induced to produce a few thoughts with reference, first, to their liability to decay, and second, as to their proper treatment. I had been disposed to believe that the lower bicuspids were the teeth most likely to remain in the mouth during life, excepting only the teeth anterior to them in the lower jaw; but owing to causes over which their possessors have no control, noticeably their early eruption and consequent feeble organization, they are gradually becoming an easy prey to dental caries. By consulting the tables of John Tomes, Magitot and the late Dr. Hitchcock, concerning the relative liability of teeth to decay, we find that the first named gives, as to number of bicuspids, both upper and lower, extracted, on account of caries, 620 out of a total of 2683 extractions, or a little less than one-fourth of the whole number being bicuspids. M. Magitot, in 10,000 cases, recalls 2620 carious bicuspids, of which 1750 were upper and 870 lower; making it appear that to one decayed lower bicuspid, there was a fraction more than two of the upper ones, similarly affected, while Dr. Hitchcock, in 20,000 cases, reports 4658 of these teeth decayed, 3303 ot which were upper and 1355 lower bicuspids; attention is directed to the tables of Dr. H., because they show, that, to one of the lower nearly two and a half of the upper bicuspids are attacked by caries. Not questioning the correctness of these compilations, I venture to state the belief, that climate, habits of life, particularly as between cities and the country, indulgence of the appetite, during the producing seasons, in sugar, grape and cider districts, and the prevalence in certain portions of the country of rheumatism, malarial and other fevers, if taken into account, would modify materially (were the statistics quoted) the at present supposed preponderance of carious upper teeth, over those of the lower jaw. I present herewith, the results of my own observations respecting these teeth, when operated upon *exclusively*, for filling; these cases passed under my notice, during a period extending over five years; they comprise all nationalities, with a preponderance of Americans. In 760 cases under treatment 340 presented carious upper and 264 carious lower bicuspids. Total number of teeth requiring treatment in the upper jaw 681, while in the lower jaw there were 507. The way in which the decays can be shown is as follows: In 45 cases 4, 48 cases 3, 110 cases 2, and in 137 cases 1 upper bicuspid had to be filled; in 26 cases 4, 37 cases 3, 90 cases 2, and in 112 cases 1 lower bicuspid was filled. Taking the total number of teeth that were filled, it may be seen, that, with regard to the relative liability of teeth to decay, my little table does not correspond with the ones above presented. It is true that very many more dentures are constructed for the upper than for the lower jaw; but this fact of itself does not prove that it was necessary on account of caries, for those teeth to have been extracted. It is a well known fact, that many persons, of their own accord, and others following the advice of cheap base-workers, suffer the loss of the upper teeth, and at the same time are extremely solicitous about saving the lower ones, fancying, from the experience of others, that it would be troublesome, and sometimes, almost impossible, to wear comfortable a lower denture; again, it is only necessary for a practitioner of long experience to draw on his recollection to find numerous instances where he has been importuned to extract *for the upper jaw* teeth that were sound, but irregularly arranged, and on his refusal to do so persons have sought less scrupulous hands, where such requests are readily acceded to.

These, and various other circumstances, in addition to the results shown by my records, and a close observation of teeth upon which I have not made operations, lead me to express the belief that the lower bicuspids are not very much less liable to decay than the upper ones ; taking this view of the subject, the question of their proper treatment is not an inappropriate one. There are a few things which should be scrupulously attended to, preliminary to filling, not these teeth alone, but whenever a considerable number of fillings is to be inserted, among which, is the removal of adjacent or other badly decayed roots of teeth, also all deposits of salivary calculus; and if there be any abnormal condition of the gums, other than that superinduced by the above causes, it should also be corrected either before or during the operation of filling. After these initial steps are taken one other thing may be done, for the comfort of the patient, by filling temporarily the larger cavities in these teeth, for from one to three weeks, or longer. When the necks of these teeth are sensitive, and the secretions apparently normal, much benefit will be derived from a rigorous polishing, using a wooden point dipped into a paste of alcohol and precipitated chalk. In the mouths of the young, I treat incipient caries with a disc, and polish and repolish, from time to time, until I am satisfied that decay will not recur. There can be no excuse in these times for neglecting to discover the presence of caries. A thorough examination of the teeth, though it be difficult of accomplishment, will save yourself from after humiliation, and the patient from much pain, and possibly the loss of a tooth. A paper was read at the recent meeting of the American Dental Association, in Chicago, by Dr. L. C. Ingersoll, of Keokuk, Iowa, entitled, "Is the Dental Pulp essential to the integrity of the Dental Structure?" I have not the time, or space, or inclination, at present, to attempt an answer to the above query, but will say, that I believe the dental pulp *is* essential to the integrity of the dental structure, and especially so with reference to the lower bicuspids. An attempt should be made to save the pulps of these teeth, if for no other reason than that it is their normal condition to be supplied with them. It seems to me that no argument is necessary to sustain a position in favor of saving instead of destroying the pulp of a tooth. Mine is a flexible rule, on the point of capping an exposed pulp. I have had more success by using some substance between the pulp and oxy-chloride of zinc, than when it was applied to the point of exposure, *after* the pulp was ba thed in creosote. One thing should be kept in mind, that is to use a material non-conducting, hard, and one which will not occupy much space when in position.

If the pulp is already dead or dying, nothing can be done except to treat and fill the root. It is not as easy to break up an abscess on a lower bicuspid root as upon an upper one ; this is explained in part by the position of these teeth in the mouth, many of them leaning inward and forward, making it a matter of some difficulty to enter the root canal, especially if the cavity is situated on the distal surface of the tooth. My own practice when dealing with pulpless lower bicuspids is, no matter by what method, to cure all that are abscessed, and fill the roots perfectly (if possible,) with an indestructible material.

" To separate or not to separate " is the question. If the molars are present, I generally make separations, patterned after Arthur's system, but when they are all gone, and the bicuspids are of good structure, it is better in my opinion to contour them. If a patient is careless about cleaning his teeth, he will receive more benefit at the hands of a dentist who permanently separates them than he will if they are restored to their natural shape. One reason in support of this is, that he will be compelled to use a toothpick, or floss silk to be comfortable after eating.

The question of permanently separating the teeth, in a measure limits our choice of materials for permanent fillings to two, as we cannot rely upon tin for contour work. After filling the root of a lower bicuspid tooth, I consider that all or nearly all, of the preliminary labor is thrown away, if the cavity be filled with amalgam. We need not hope to preserve such teeth for a longer period than five years, if they are so treated. When we find small proximal, or labial cavities in these teeth, I believe with Prof. Judd, that "as it is not the amount of material used but the time consumed in performing the operation, for which a charge is made," we ought to fill such cavities with gold, instead of tin, or amalgam; however, there are teeth which, when filled with tin, remain free from caries, as well as those tha are filled with gold; but its use is limited to proximal cavities mainly and then I prefer to make wide separations before using it. When a case is presented to my notice, where these teeth have not been filled, I cannot persuade myself that it will be to the best interest of the patient to fill them with amalgam. If we fill the crown cavity of a lower bicuspid tooth with amalgam, we cannot be sure that in a few months or a year or two, there will not be a labial or proximal cavity in the same tooth; and in the event of such being the case, we are compelled, either to remove the first filling, and fill the cavities with gold, or insert another filling with amalgam. Would it not have been wiser to fill the crown cavities with gold in the first place?

Avoiding all reference to the appearance of teeth filled with amalgam, and the discolorations coincident with its use therein, and its known liability to shift after being placed in the cavity (all very serious objections, especially when used anterior to the molars,) what brand of amalgam in this or any other country, that will exclude air and moisture from a cavity in a tooth as perfectly as gold? Until a non-shrinkable, and a non-oxydizable plastic filling material is discovered, we cannot hope to obtain results at all comparable to those which are to be secured by the use of gold.

To sum up my idea about the treatment of the lower bicuspids, would be to say: use, if you would be successful in filling these teeth, gold first, tin second, and amalgam rarely if ever—*Missouri Dental Journal.*

